# An observational study of the correlation of efi severity with depression

**DOI:** 10.1192/j.eurpsy.2022.1669

**Published:** 2022-09-01

**Authors:** R. Rasheed, A. Patel, Y. Shanthakumaran

**Affiliations:** Rigg Milner Medical Centre, Gp Education And Research, East Tilbury, United Kingdom

**Keywords:** Depression, Frailty, Electronic Frailty Indices

## Abstract

**Introduction:**

Patients with high fraility indices experience poor mental health due to multiple co morbidity and social isolation.

**Objectives:**

This was a retrospective observational analysis that studied the correlation of Electronic frailty indices and GAD scores with Depression scores in a rural population.

**Methods:**

An annual frailty assessment is offered to elderly patients and we screen routinely for anxiety and depression using the PHQ-9 score and GAD score. This was an observational study examining the correlation of the Electronic Frailty Indices (EFI) depression and anxiety scores.

**Results:**

Of the 118 patients ranging from mild to severe frailty we found a positive correlation of the EFI with the Depression and anxiety scores. Within the data set, the correlation coefficient of EFI scores and PHQ 9 scores was found to be 0.819. Similarly within the same data set we found a correlation coefficient of EFI and GDS scores of 0.651. The higher the EFI the greater was the scale of dependency and comorbidity and this correlation was consistent across the data set with depression and anxiety. We believe physical impairment, loss of independence and social isolation cognitive decline contribute to loss of self-esteem.

**Conclusions:**

Our study found a positive correlation between frailty severity based on EFI scores and depression and anxiety severity. Early detection in deterioration of mental health will enable supportive measures and targeted treatment strategies. Our study shows the strong correlation of EFI severity scores with worse mental health.

**Disclosure:**

No significant relationships.

